# Altered Neuromagnetic Activity in Persistent Postural-Perceptual Dizziness: A Multifrequency Magnetoencephalography Study

**DOI:** 10.3389/fnhum.2022.759103

**Published:** 2022-03-08

**Authors:** Weiwei Jiang, Jintao Sun, Jing Xiang, Yulei Sun, Lu Tang, Ke Zhang, Qiqi Chen, Xiaoshan Wang

**Affiliations:** ^1^Department of Neurology, The Affiliated Brain Hospital of Nanjing Medical University, Nanjing Medical University, Nanjing, China; ^2^Division of Neurology, MEG Center, Cincinnati Children’s Hospital Medical Center, Cincinnati, OH, United States

**Keywords:** persistent postural-perceptual dizziness, magnetoencephalography, cortical dysfunctions, multifrequency bands, accumulated source imaging

## Abstract

**Objective:**

The aim of our study was to investigate abnormal changes in brain activity in patients with persistent postural-perceptual dizziness (PPPD) using magnetoencephalography (MEG).

**Methods:**

Magnetoencephalography recordings from 18 PPPD patients and 18 healthy controls were analyzed to determine the source of brain activity in seven frequency ranges using accumulated source imaging (ASI).

**Results:**

Our study showed that significant changes in the patterns of localization in the temporal-parietal junction (TPJ) were observed at 1–4, 4–8, and 12–30 Hz in PPPD patients compared with healthy controls, and changes in the frontal cortex were found at 1–4, 80–250, and 250–500 Hz in PPPD patients compared with controls. The neuromagnetic activity in TPJ was observed increased significantly in 1–4 and 4–8 Hz, while the neuromagnetic activity in frontal cortex was found increased significantly in 1–4 Hz. In addition, the localized source strength in TPJ in 1–4 Hz was positively correlated with DHI score (*r* = 0.7085, *p* < 0.05), while the localized source strength in frontal cortex in 1–4 Hz was positively correlated with HAMA score (*r* = 0.5542, *p* < 0.05).

**Conclusion:**

Our results demonstrated that alterations in the TPJ and frontal cortex may play a critical role in the pathophysiological mechanism of PPPD. The neuromagnetic activity in TPJ may be related to dizziness symptom of PPPD patients, while the neuromagnetic activity in frontal lobe may be related to emotional symptoms of PPPD patients. In addition, frequency-dependent changes in neuromagnetic activity, especially neuromagnetic activity in low frequency bands, were involved in the pathophysiology of PPPD.

## Introduction

Persistent postural-perceptual dizziness (PPPD) is considered a chronic functional vestibular disorder with clinical symptoms and has been defined by the World Health Organization (WHO) and the Bárány Society (Staab et al., 2017). The clinical characteristics of PPPD were persistent dizziness and/or unsteadiness for more than 3 months. Several factors, such as active/passive motion, upright posture, and exposure to a complex environment, can aggravate the symptoms of PPPD (Staab et al., 2017). A previous study demonstrated that PPPD was one of the most common causes of chronic vestibular symptoms (Dieterich and Staab, 2017). However, the potential pathogenesis of PPPD remains unclear and has received much attention from clinical researchers.

Importantly, postural unsteadiness and visual dizziness are the core features of PPPD (Sun and Xiang, 2020). Patients with PPPD adopt a stiffened postural control strategy in response to dizzying trigger events and persistently maintain this adaptation even if the trigger factors disappear (Cao et al., 2021). In addition, researchers found that the ability to control posture through multisensory information inputs was impaired in patients with PPPD (Sohsten et al., 2016). PPPD patients were more dependent on visual or somatosensory inputs than vestibular inputs to control their body posture (Staab et al., 2017). Thus, cortical integration dysfunction involving multisensory inputs may be suggested as a potential pathophysiological mechanism of PPPD.

Recently, with the development of neuroimaging techniques, several neuroimaging methods have been used to investigate alterations in brain function in patients with PPPD. Two studies investigating structural alterations in the brain in PPPD patients using magnetic resonance imaging (MRI) found that both gray matter volume and structural networks were changed in specific brain regions (Wurthmann et al., 2017; Popp et al., 2018). Another study using single-photon emission computed tomography (SPECT) to explore regional cerebral blood flow (rCBF) in PPPD patients showed that the changes in rCBF were significantly different in several brain regions (Na et al., 2019). Moreover, several studies investigating brain activity in PPPD patients using functional MRI (fMRI) showed that the functional connectivity was altered in brain regions involved in the processing of multisensory vestibular information (Lee et al., 2018; Li et al., 2020b). In sum, the above studies indicated that cortical dysfunction could be observed in patients with PPPD using neuroimaging methods.

Magnetoencephalography (MEG) is a relatively new non-invasive technique that can be used to detect neuromagnetic signals. Recently, MEG was used to study epilepsy, migraine, and schizophrenia (Liu et al., 2015; Tang et al., 2016; Wu et al., 2016; Lottman et al., 2019; Sun et al., 2020) and used in the clinical evaluation of epileptogenic foci before epileptic surgery (De Tiege et al., 2017; Tamilia et al., 2017). MEG has a higher spatial resolution than electroencephalography (EEG) and can detect magnetic signals that are unaffected by the skull and skin (Babiloni et al., 2009). Moreover, MEG can detect signals with time resolution of milliseconds in wider frequency ranges (Moradi et al., 2003; Babiloni et al., 2009). Given the abovementioned findings, MEG could be an ideal tool to investigate cortical dysfunction in PPPD patients.

The aim of the present study was to investigate the abnormal spatial changes in brain activity in patients with PPPD. In this investigation, neuromagnetic signals were analyzed in low- to high-frequency bands using MEG. To the best of our knowledge, our study is the first to explore abnormal spatial alterations in brain activity in patients with PPPD using MEG. The investigation of alterations in brain function in patients with PPPD could improve the present understanding of the pathogenesis of PPPD and contribute to the development of treatments for PPPD in the future.

## Materials and Methods

### Subjects

Eighteen right-handed patients diagnosed with PPPD were enrolled from Nanjing Brain Hospital. The inclusion criteria were as follows: (1) the clinical diagnosis of PPPD was in line with the diagnostic criteria of the Classification Committee of the Bárány Society (Staab et al., 2017), (2) no history of medication before enrollment, and (3) no abnormal MRI results. The exclusion criteria were as follows: (1) PPPD coexisting with peripheral vestibular lesions or other diseases and (2) the presence of metal implants in the head. In addition, eighteen age- and sex-matched healthy volunteers were included as controls. All healthy volunteers had no history of headache, dizziness, or other serious medical diseases. The present study was approved by the Medical Ethics Committees of Nanjing Brain Hospital and Nanjing Medical University. Informed assent was signed by all subjects. All methods were performed in accordance with the relevant guidelines and regulations of the Declaration of Helsinki for human experimentation.

### Magnetoencephalography Recording

Magnetoencephalography data were recorded by a whole-head CTF 275 Channel MEG system (VSM Medical Technology Company, Coquitlam, BC, Canada) in a magnetic-shielded room in the MEG Center of Nanjing Brain Hospital. Background noise was identified by recording MEG signals in an empty room. Before formal MEG data recording, each subject was required to remove all metals from his or her body. Then, three coils were attached to the nasion and to the right and left preauricular points of each subject to locate the subject’s position head relative to the MEG recording system. During MEG recording, each subject was asked to stay still with his or her eyes closed slightly and was monitored by an audio-visual system from an MEG recording device. At least five continuous MEG data files with a duration of 120 s were recorded for each subject. Each subject’s head movement was limited to 5 mm for each MEG recording. The MEG data were collected at a sample of 6,000 Hz, with noise cancelation of third-order gradients.

### Magnetic Resonance Imaging Scans

A 3.0-T MRI scanner (Siemens, Germany) was used to acquire three-dimensional structural images. A rapid gradient echo sequence was used to obtain anatomic 3D T1-weighted images. The imaging parameters were as follows: field of view 250 mm × 250 mm; flip angle 9°; matrix = 512 × 512. A total of 176 sagittal slices were collected for each subject. All subjects were required to minimize their head movements during MRI scanning. For each subject, markers were placed on the same three fiducial positions used for MEG recording to coregister the MRI data with the MEG data.

### Data Analysis

For each MEG segment, magnetic noise and artifacts were identified by visual inspection. MEG segments without any artifacts and noise were viewed as clean MEG data and selected for further analysis. The duration of each clean MEG segment for further analysis was 100 s. The selected segments were analyzed in seven frequency bands: delta (1–4 Hz), theta (4–8 Hz), alpha (8–12 Hz), beta (12–30 Hz), gamma (30–80 Hz), ripple (80–250 Hz), and fast ripple (250–500 Hz). Notch filters for 50 Hz and its harmonics were performed to eliminate power-line noise from the MEG data.

Accumulated source imaging (ASI), which was defined as the volumetric summation of source activity over a period of time, was used to localize neuromagnetic activity (Xiang et al., 2014). The neuromagnetic sources were localized by ASI using node-beam lead fields (Xiang et al., 2014). Since each node-beam lead field indicated a form of either source-beamformer or subspace solution, the ASI had multiple source beamformers to separate correlated sources. Several previous studies have verified the reliability of this method (Xiang et al., 2014, 2015a,b; Liu et al., 2015; Tang et al., 2016; Li et al., 2020c; Sun et al., 2020).

Similar to previous studies (Xiang et al., 2009; Tang et al., 2016; Sun et al., 2020), we analyzed neuromagnetic activity based on individual MRI head models at the source level. The whole brain was scanned at 3 mm resolution (approximately 17,160 brain voxels, depending on the size of the brain) (Xiang et al., 2014). Volumetric source imaging was computed for each frequency band for each MEG data. The formula was defined as follows:


(1) $$\text{Asi}\,\left(\text{r},\text{s}\right)=\sum_{\text{t}=1}^{\text{t}=\text{n}}\text{Q}\,\left(\text{r},\text{t}\right)$$

In Equation (1), Asi represents the accumulative source strength at location r, s indicates the time slice, t represents the MEG data point in the selected slice s, Q indicates the source activity at location r and time point t, and n represents the number total time points included in the MEG data. Detailed mathematical algorithms and validations were demonstrated in previous publications (Xiang et al., 2014, 2015a). In addition, each voxel of source imaging contained a specific parameter to quantify neuromagnetic activity strength. Since the source strength was analyzed statistically, no units were provided for parameters (Xiang et al., 2015a). MEG source imaging was coregistered to MRI according to the three fiducial points used for MEG recording and then normalized spatially for group analyses.

### Statistical Analysis

Fisher’s exact test was used to identify the difference in neuromagnetic source locations between patients with PPPD and healthy controls. Student’s *t*-test was performed to compare source strength between two groups. Partial correlation was applied to estimate the correlations between the source strength and clinical sores after adjustment for sex and age. The *p*-value threshold in the present study was defined as 0.05. A false discovery rate controlling procedure was used to solve type I errors. Bonferroni correction was used for multiple comparisons. Statistical analyses and computations were performed in SPSS version 20.0 for Windows (SPSS Inc., Chicago, IL, United States).

## Results

### Clinical Characteristics

Eighteen patients with PPPD and eighteen healthy controls were enrolled in this study. The Dizziness Handicap Inventory score (DHI), the Hamilton Anxiety scale (HAMA), and the Hamilton Depression scale (HAMD) were applied to assess symptoms in patients with PPPD. The average age of PPPD patients was 55.39 ± 12.52 years. The mean duration of disease was 8.17 ± 4.38, mean DHI score was 52.72 ± 7.4, mean HAMA score was 16.61 ± 4.23, and mean HAMD was 13.44 ± 5.07. The detailed characteristics of PPPD patients are shown in Table 1. A total of 18 MEG segments from patients and 18 MEG segments from healthy controls were selected for further analysis.

**TABLE 1:** Characteristics of the patients with PPPD.

Patient	Sex (F/M)	Age (years)	Duration of disease (months)	DHI	HAMA	HAMD	Dizziness	Unsteadiness	Non-spinning vertigo
1	F	79	5	54	11	9	+	+	+
2	M	39	12	62	18	17	+	+	+
3	F	63	8	48	16	12	+	+	+
4	F	49	3	44	10	7	+	+	+
5	F	42	14	57	20	20	+	+	+
6	F	57	9	49	16	11	+	+	+
7	F	70	4	39	13	6	+	+	+
8	F	48	7	52	17	10	+	+	
9	M	57	4	57	15	13	+	+	+
10	F	54	2	55	18	16	+	+	+
11	M	51	7	49	12	9	+	+	+
12	F	50	9	58	19	15	+	+	+
13	F	57	15	66	26	24	+	+	+
14	F	54	18	64	24	21	+	+	
15	F	75	10	51	16	18	+	+	+
16	F	33	4	47	19	11	+	+	+
17	M	47	8	42	12	9	+	+	
18	F	72	8	55	17	14	+	+	

*F, female; M, male; DHI, Dizziness Handicap Inventory score; HAMA, Hamilton Anxiety scale; HAMD, Hamilton Depression scale. ^+^indicates symptoms exist.*

### Source Localization Pattern

**FIGURE 1:**
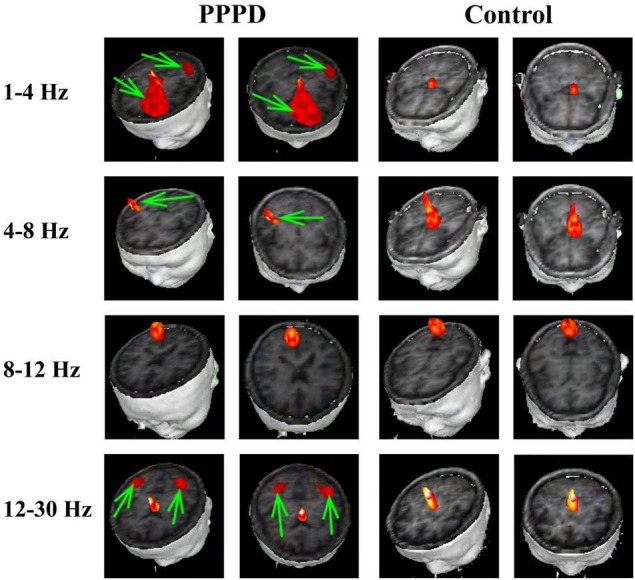
Typical distribution of neuromagnetic source localization in 1–30 Hz frequency bands in PPPD patients and controls. Green arrows indicate the significant difference in source localization in PPPD patients compared with the controls.

**FIGURE 2:**
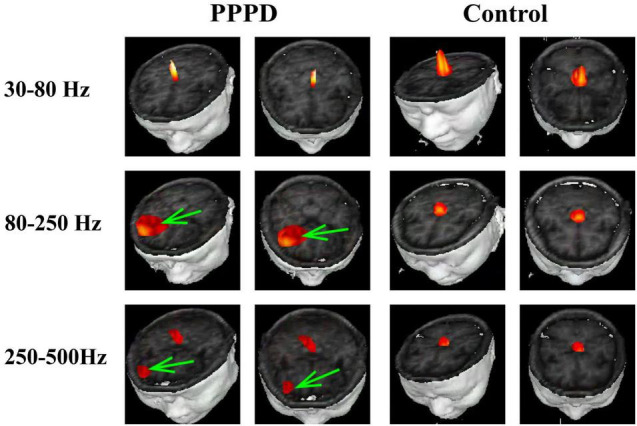
Typical distribution of neuromagnetic source localization in 30–500 Hz frequency bands in PPPD patients and controls. Green arrows indicate the significant difference in source localization in PPPD patients compared with the controls.

**FIGURE 3:**
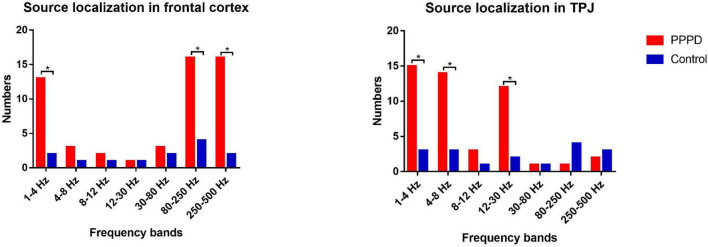
Difference in the number of neuromagnetic source localization between PPPD patients and the controls in seven frequency bands. The number of source locations is located on the *y*-axis. Seven frequency bands are listed on the *x*-axis. *p < 0.05 after Bonferroni correction.

At 1–4 Hz, the neuromagnetic source was mainly localized in the frontal cortex and temporal-parietal junction (TPJ) (7 in right TPJ, 4 in left TPJ, and 4 in bilateral TPJ) in patients with PPPD compared with that in healthy controls (*p* < 0.05). At 4–8 Hz, neuromagnetic activity was mainly localized in the TPJ (5 in right TPJ, 5 in left TPJ, and 4 in bilateral TPJ) in patients with PPPD compared with that in healthy controls (*p* < 0.05). At 8–12 Hz, neuromagnetic activity was mainly localized in the occipital cortex in both PPPD patients and healthy controls (*p* > 0.05). At 12–30 Hz, neuromagnetic activity was mainly localized in the TPJ (4 in right TPJ, 3 in left TPJ, and 5 in bilateral TPJ) in patients with PPPD compared with that in healthy controls (*p* < 0.05). In 30–80 Hz, neuromagnetic activity was mainly localized in deep brain areas (DBA) in both PPPD patients and healthy controls (*p* > 0.05). In the high-frequency ranges (80–250 and 250–500 Hz), PPPD subjects had a significantly higher odds ratio in the frontal cortex than healthy controls (*p* < 0.05). See Figures 1–3 for details.

### Source Strength in Neuromagnetic Activity

**FIGURE 4:**
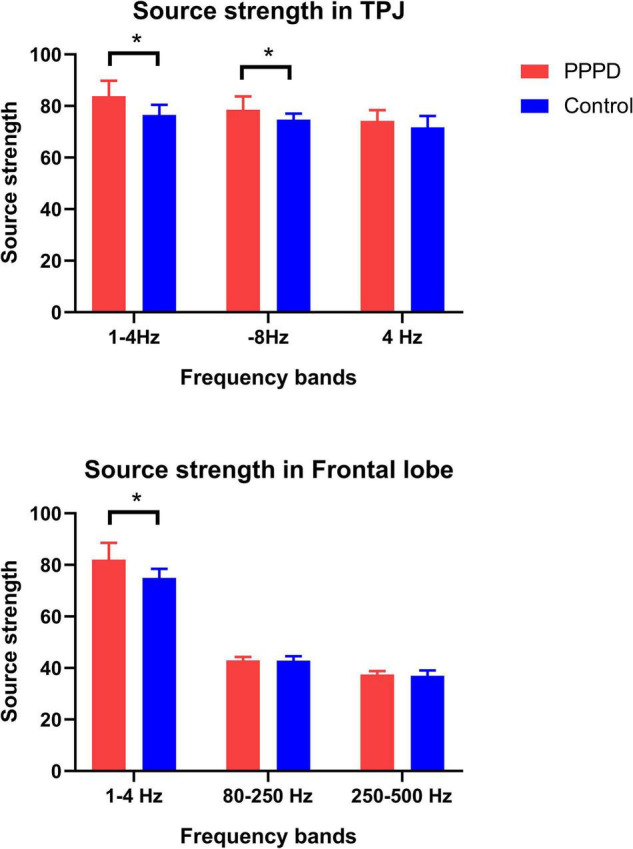
Changes in neuromagnetic source strength in specific brain regions between two groups. *P < 0.05 after Bonferroni correction.

There was no significant difference in source strength in whole brain level between patients with PPPD and controls in all seven frequency bands (Table 2). However, significant changes in the localized source strength of specific brain areas were observed in low frequency bands between PPPD patients and healthy controls. In 1–4 Hz, the localized source strength in TPJ and frontal cortex in PPPD was significantly higher than corresponding brain regions in controls (*p* < 0.05). In 4–8 Hz, the localized source strength in TPJ in PPPD was significantly higher than healthy controls (*p* < 0.05). See Figure 4 for details.

**TABLE 2:** Comparison of neuromagnetic source strength between PPPD patients and controls.

Frequency bands	PPPD patient	Control	*P* value
1–4 Hz	84.29 ± 5.99	84.45 ± 8.61	*P* > 0.05
4–8 Hz	78.65 ± 5.39	79.58 ± 5.55	*P* > 0.05
8–12 Hz	82.24 ± 12.79	84.03 ± 17.03	*P* > 0.05
12–30 Hz	74.51 ± 4.68	74.79 ± 7.62	*P* > 0.05
30–80 Hz	57.39 ± 6.97	54.88 ± 9.17	*P* > 0.05
80–250 Hz	43.27 ± 1.80	43.52 ± 2.15	*P* > 0.05
250–500 Hz	38.29 ± 1.02	38.59 ± 2.54	*P* > 0.05

*There was no significant difference between two groups after Bonferroni correction.*

### Correlations Between the Source Strength and Clinical Scores

**FIGURE 5:**
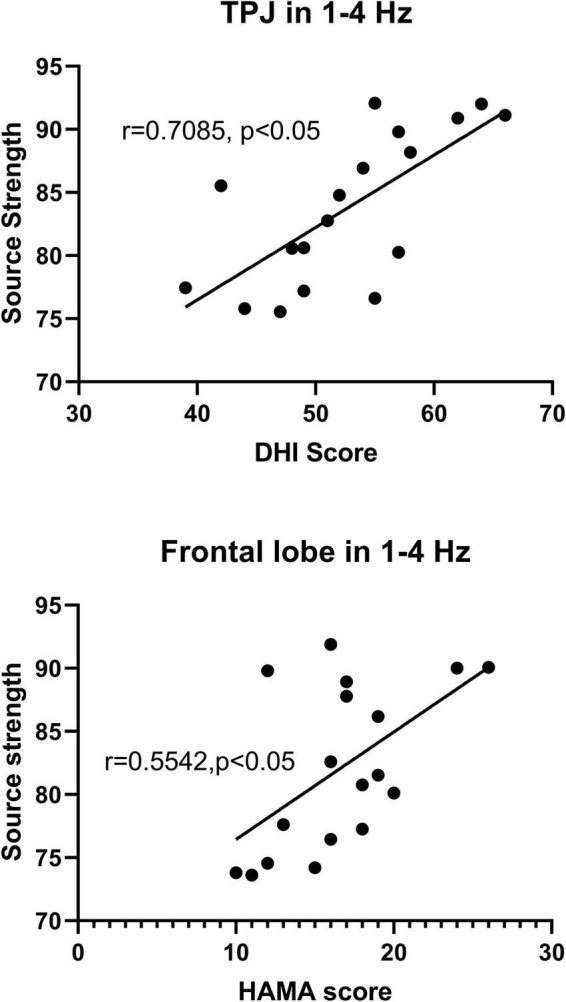
The *y*-axis represents the neuromagnetic source strength. The *x*-axis represents clinical scores. Partial correlation analysis showed that the neuromagnetic source strength in TPJ in 1–4 Hz was positively correlated with DHI score (*r* = 0.7085, *p* < 0.05), and the neuromagnetic source strength in frontal cortex in 1–4 Hz was positively correlated with HAMA score (*r* = 0.5542, *p* < 0.05) after adjustment for sex and age.

In the present study, significant correlations were observed between the localized source strength in specific brain regions in low frequency band and clinical scores in PPPD patients. Specifically, in 1–4 Hz, the localized source strength in TPJ was positively correlated with DHI score (*r* = 0.7085, *p* < 0.05), while the localized source strength in frontal cortex was positively correlated with HAMA score (*r* = 0.5542, *p* < 0.05). No significant correlation was observed between the local source strength and clinical scores in other frequency bands. The details are shown in Figure 5.

## Discussion

In the present study, we explored cortical neuromagnetic activity in patients with PPPD by multifrequency analysis using MEG. The results showed that frequency-dependent alterations in neuromagnetic activity were observed in patients with PPPD compared with healthy controls. To the best of our knowledge, this is the first study to investigate neuromagnetic activity in PPPD patients using MEG.

Our results found that the abnormal neuromagnetic source localization in patients with PPPD was more likely to be located in the TPJ than in healthy controls, indicating that the function of this brain region was disordered in patients with PPPD. Our study also founded that the neuromagnetic source strength in TPJ in PPPD patients was significantly higher than healthy controls indicating that the neural activity of TPJ in PPPD patients was increased abnormally. In addition, in our research, a positive correlation between neuromagnetic source strength in TPJ and DHI score shown that the abnormal activity of TPJ may be related to the dizziness symptom of PPPD patients. Therefore, we speculate that the abnormal increase of neural activity in TPJ might be a potential factor contributing to dizziness symptom of PPPD patients. This finding was consisted with several studies. A study using resting-state fMRI showed that functional connectivity in the superior temporal gyrus (STG) was decreased in PPPD patients compared to healthy controls (Van Ombergen et al., 2017; Lee et al., 2018). Another study using task-based fMRI reported that functional connectivity was decreased between the STG and other brain regions in patients with PPPD (Indovina et al., 2015). In addition, a structural MRI study found decreased gray matter volume in the STG and parietal cortex in PPPD patients (Wurthmann et al., 2017). According to previous studies, the TPJ, including adjacent areas of the temporal and parietal lobes, is an important component of the parieto-insular vestibular cortex (PIVC) and plays a critical role in the integration and processing of multisensory information (Eickhoff et al., 2006; Wurthmann et al., 2017). A previous study found that the TPJ has bidirectional connections with frontal and temporal lobes and receive the sensory inputs from subcortical, visual and auditory areas (Decety and Lamm, 2007). The TPJ participates in motor and posture control by transmitting multisensory information to motor areas based on connections between posterior brain regions and the frontal cortex (Hoshi and Tanji, 2007). In addition, vestibular and visual sensory information are also integrated and processed via the TPJ (Takeuchi et al., 2018). A neuroimage study found that the abnormal activation was observed in TPJ when sensory integration conflict existed (Balslev et al., 2005). A previous study reported that patients whose lesions were localized in the TPJ presented with deficits in vestibular spatial orientation, also suggesting an association between the TPJ and the processing of vestibular spatial information (Kaski et al., 2016). In addition, dysfunction of the TPJ was also a potential pathophysiological mechanism of virtual reality sickness, a disease which was involved in vestibular-visual information mismatch (Takeuchi et al., 2018). Thus, we speculate that dysfunction in the TPJ weakens PPPD patients’ ability to process and integrate vestibular spatial inputs and causes them to experience persistent dizziness and unsteadiness.

Furthermore, in our study, abnormal brain activity in the frontal cortex was also observed in patients with PPPD compared with healthy controls. We founded that the neuromagnetic source strength in frontal cortex was significantly higher than controls indicating that the abnormal neural activity of frontal cortex might be another potential pathological imaging feature of PPPD patients. This finding was in line with previous literature. Several studies confirmed that brain activity in the frontal cortex was altered in PPPD patients (Indovina et al., 2015; Van Ombergen et al., 2017; Lee et al., 2018; Li et al., 2020b). One study using structural MRI also found that gray matter volume was decreased in the frontal lobe in PPPD patients, suggesting that these individuals have a structural impairment in the frontal lobe (Wurthmann et al., 2017). Moreover, a SPECT study showed hypoperfusion in the frontal lobe in PPPD patients (Na et al., 2019). Pertinently, the frontal lobe participates in diverse cortical functions, including executive function, motivation, and working memory processing, in the brain, as reported by previous publications (Pochon et al., 2001; du Boisgueheneuc et al., 2006). The motor regions of the frontal cortex regulate posture and complex movements by receiving information from sensory regions of the parietal cortex (Wurthmann et al., 2017). Therefore, when the TPJ is dysfunctional, the motor areas of the frontal lobe cannot obtain accurate multisensory information from the external environment and patients’ ability to regulate their body posture is affected. In addition, the frontal cortex is involved in emotion modulation (Lee et al., 2018). Previous studies reported that functional alterations in the frontal cortex were observed in patients with several psychiatric diseases, such as schizophrenia and autism spectrum disorder (Xiang et al., 2016; Lee et al., 2017). An fMRI study found that alterations in the frontal cortex were not observed after adjusting for anxiety and depression in patients with PPPD, indicating that abnormal changes in the frontal cortex appeared to be related to coexisting pathological psychiatric states, such as anxiety and depression, in PPPD patients (Lee et al., 2018). In our study, we founded that the neuromagnetic source strength in frontal cortex was positively correlated with HAMA score suggesting that the abnormal increase in neural activity of frontal cortex may be related to emotional symptom of PPPD patients. Our results indicated that emotional symptoms of PPPD patients, such as anxiety, may be resulted from a potential pathological basis in the frontal lobe, not just emotional feedback caused by dizziness symptoms of PPPD patients. Thus, we speculate that the functional impairment of the frontal lobe plays an important role in postural balance disturbance and emotional disorders in patients with PPPD.

For the first time, our study used MEG to demonstrate frequency-dependent alterations in cortical localized patterns in PPPD patients. We found that alterations in cortical localized patterns in the TPJ were observed in low-frequency bands (mainly in 1–4, 4–8, and 12–30 Hz), and alterations in the frontal cortex were observed in both low- and high-frequency bands (mainly in 1–4, 80–250, and 250–500 Hz). However, it is worth noting that the significant increase in neuromagnetic source strength of specific brain areas in PPPD patients compared with healthy controls was observed in low frequency (mainly in 1–4 and 4–8 Hz), while in other frequency bands the significant changes in the localized neuromagnetic source strength of specific brain regions between two groups were not founded. According to previous studies, frequency-dependent changes in brain activity were observed in patients with other diseases, such as epilepsy, migraine, schizophrenia, and autism spectrum disorder (Tenney et al., 2014; Xiang et al., 2015a; Wu et al., 2016; Lee et al., 2017). As reported by several studies, the types of connections and information interactions at the temporal-spatial level among neurons were different in different frequency bands (Tenney et al., 2014). It was presumed that the speed of information transmission was restricted by the frequency of oscillations. In lower frequency bands, oscillations were suited to integrate information from large regions (Sauseng and Klimesch, 2008). However, in higher frequency ranges, oscillations were used to interact with neighboring neurons (Engel and da Silva, 2012). The research mentioned above could partially explain our findings in different frequency bands. We speculate that the function of information integration in large brain regions in lower frequency bands could support the processing and integration of sensory information from the external environment in the TPJ. The abnormal changes of neuromagnetic activity involving TPJ and frontal lobe in low frequency bands may be an important pathological imaging feature of PPPD. However, the specific mechanism underlying frequency-dependent changes found in the present study should be further investigated in the future.

Abnormal changes in cortical function were observed in patients with PPPD in fMRI studies (Indovina et al., 2015; Li et al., 2020a). To our knowledge, the basis of neuronal activity in the brain is electrical activity. The electrical activity of the brain in cell level is accompanied by changes in the magnetic field leading to corresponding changes in the neuromagnetic signal. Therefore, we believe that the cortical dysfunction in PPPD could cause changes in neuromagnetic signals in specific brain regions, which can be detected by MEG. In addition, a previous study using EEG have found that patients with dizziness can find abnormal brain activity in the low frequency bands, which suggested that although there is no epileptic activity in patients with dizziness, other forms of abnormal brain activity could be detected by EEG, such as abnormal brain activity in the low frequency bands (Nürnberger et al., 2021). MEG has higher spatial resolution than EEG and the magnetic signal is not affected by skull and skin. Therefore, using MEG to analyze the brain activity in patients with PPPD can get more accurate and reliable results than EEG. Source localization is an analytical method used to quantify neuromagnetic signals in the brain (Xiang et al., 2014, 2015b). By this method, subtle brain activity can be detected and located in the cortical region. We believe that using this method, we can accurately detect subtle changes in neural activity of specific brain regions and localize the cortical pathological changes in PPPD patients. In addition, source localization can also provide quantitative parameters to evaluate the subtle changes in cortical function, which could provide an objective basis for the study of the relationship between specific cortical functions and clinical symptoms. Through this method, we have studied several kinds of diseases, such as childhood absence epilepsy, migraine and so on, and obtained several localization and quantitative research results (Liu et al., 2015; Tang et al., 2016; Sun et al., 2020). Therefore, we think that the application of MEG and source localization method could obtain ideal results in PPPD research.

### Limitations

There were several limitations in our study. First, the sample size of the present study is relatively small and might influence the results in our study. A larger sample size of study could be necessary to further validate our research in the future. Second, MEG is not sensitive to magnetic signals in DBA. Therefore, we only analyzed and discussed the changes in cortical functions in PPPD patients. With the application of wearable MEG devices, the above problem can be resolved. Third, due to the limitation of software, we did not analyze, and process negatively activated brain regions in patients with PPPD. Therefore, we only discussed the results of activated brain regions localized by MEG in this paper. With the progress of software technology, this problem could be solved in the future. Fourth, in this study, we found neuromagnetic localization in the right, left and bilateral TPJ at 1–4, 4–8, and 12–30 Hz. However, there was no significant difference among these locations of TPJ. We believe that the sample size could be the reason for the above results. In the future study, with the further expansion of sample size, we will be able to further understand the location characteristics of TPJ in those frequency bands. Although frequency-dependent changes were found in the present study, we also believed that the above results were only preliminary conclusion, and the specific relationship between these changes and PPPD needs further research using other methods to explore. In addition, we also agree that the study of brain networks in patients with PPPD can further deepen the understanding of the pathophysiological mechanism of PPPD. Indeed, the analysis of abnormal neuromagnetic localizations in patients with PPPD was a preliminary study of functional imaging of PPPD. Therefore, in the future, we will further investigate the brain network of PPPD based on the results of neuromagnetic source localization in PPPD patients in the present study, in order to further explore the pathophysiological mechanism of PPPD. Finally, although various methods were used to minimize artifacts, our results may still have been affected by residual artifacts. Further research is needed to verify whether the artifacts have been completely eliminated.

## Conclusion

For the first time, our study using MEG demonstrated that abnormal changes in neuromagnetic localization in the TPJ and frontal lobe were found in low- and high-frequency bands in patients with PPPD. The localized neuromagnetic activity in TPJ and frontal cortex was abnormally increased in low frequency bands in PPPD patients compared with healthy controls. In addition, the neuromagnetic activity in TPJ may be related to dizziness symptom of PPPD patients, while the neuromagnetic activity in frontal lobe may be related to emotional symptoms of PPPD patients. These findings suggest that the TPJ and frontal lobe may play a critical role in the pathophysiological mechanism of PPPD. Our results could provide novel insights into the pathophysiology of PPPD.

## Data Availability Statement

The original contributions presented in the study are included in the article/supplementary material, further inquiries can be directed to the corresponding author.

## Ethics Statement

The studies involving human participants were reviewed and approved by the Medical Ethics Committees of Nanjing Brain Hospital and Nanjing Medical University. The patients/participants provided their written informed consent to participate in this study.

## Author Contributions

JS, WJ, JX, and XW designed the research. JS, YS, and KZ analyzed the data. JS, WJ, QC, and LT recruited the participants and acquired the images. JS wrote the manuscript. XW revised the manuscript. All authors approved the final submitted version and agreed to be accountable for its content.

## Conflict of Interest

The authors declare that the research was conducted in the absence of any commercial or financial relationships that could be construed as a potential conflict of interest.

## Publisher’s Note

All claims expressed in this article are solely those of the authors and do not necessarily represent those of their affiliated organizations, or those of the publisher, the editors and the reviewers. Any product that may be evaluated in this article, or claim that may be made by its manufacturer, is not guaranteed or endorsed by the publisher.
